# Parental Intentions and Perceptions Toward COVID-19 Vaccination Among Children Aged 4 Months to 4 Years — PROTECT Cohort, Four States, July 2021–May 2022

**DOI:** 10.15585/mmwr.mm7135a2

**Published:** 2022-09-02

**Authors:** Karen Lutrick, Ashley Fowlkes, Patrick Rivers, Katherine Herder, Tammy A. Santibanez, Lindsay LeClair, Kimberly Groover, Julie Mayo Lamberte, Lauren Grant, Leah Odame-Bamfo, Maria V. Ferraris, Andrew L. Phillips, Brian Sokol, Ashley A. Lowe, Clare Mathenge, Felipe A Pubillones, Brianna Cottam, Hilary McLeland-Wieser, Krystal S. Jovel, Jezahel S. Ochoa, Jacob Mckell, Mark Berry, Sana Khan, Natasha Schaefer Solle, Ramona P. Rai, Flavia Miiro Nakayima, Gabriella Newes-Adeyi, Cynthia Porter, Zoe Baccam, Katherine D. Ellingson, Jeffery L. Burgess, Manjusha Gaglani, Lisa Gwynn, Alberto Caban-Martinez, Sarang Yoon

**Affiliations:** ^1^Family and Community Medicine, College of Medicine – Tucson, University of Arizona Health Sciences, Tucson, Arizona;^ 2^CDC COVID-19 Emergency Response Team; ^3^Mel and Enid Zuckerman College of Public Health, University of Arizona, Tucson, Arizona; ^4^Abt Associates, Rockville, Maryland; ^5^Baylor Scott & White Health, Texas A&M University School of Medicine, Temple, Texas; ^6^Leonard M. Miller School of Medicine, University of Miami, Miami, Florida; ^7^Rocky Mountain Center for Occupational and Environmental Health, Department of Family and Preventive Medicine, University of Utah Health, Salt Lake City, Utah.

Approximately 12 million children and adolescents aged ≤18 years in the United States have been infected with SARS-CoV-2, the virus that causes COVID-19, since December 2019,* and COVID-19–associated hospitalization rates increased among children aged <5 years during the B.1.617.2 (Delta) and B.1.1.529 (Omicron) variant peaks (*1*). In June 2022, the Food and Drug Administration amended the Emergency Use Authorization for the BNT162b2 (Pfizer-BioNTech) COVID-19 vaccine to include use of the vaccine in children aged 6 months–4 years and mRNA-1273 (Moderna) for children 6 months–5 years, which CDC recommends all children receive.^†^ Advance reports indicated that fewer than 50% of parents were willing to vaccinate their children aged <5 years (*2*,*3*). Using the Pediatric Research Observing Trends and Exposures in COVID-19 Timelines (PROTECT)^§^ (*4*) prospective cohort, changes in parental perceptions toward COVID-19 vaccines and vaccination^¶^ for children aged <5 years were examined during July 2021–May 2022. Among 393 parents who participated in a baseline survey, approximately 64%, 19%, and 10% reported they were likely, were unsure, or were unlikely, respectively, to have their child aged <5 years receive the COVID-19 vaccine. The odds of parents intending to vaccinate their child was lower 3 months after the baseline survey, (adjusted odds ratio [aOR] = 0.84, 95% CI = 0.6–1.0) than at baseline. During the same period, parents also were less likely to perceive that COVID-19 vaccines were effective (aOR = 0.61, 95% CI = 0.4–0.8) and safe (aOR = 0.65, 95% CI = 0.5–0.9) compared with baseline. Intent to vaccinate and perception of safety increased 6 months after the baseline survey in unadjusted models (OR = 1.66, 95% CI = 1.1–2.5; and OR = 1.82, 95% CI = 1.3–2.6, respectively), but were no longer significant after adjusting for the child’s receipt of a positive SARS-CoV-2 test result before survey completion, age, sex, race and ethnicity, health insurance, and study site. Enhanced efforts to address parental confidence in childhood vaccination and increase vaccination coverage among children aged <5 years are needed, including reinforcing the effectiveness and safety of vaccination against COVID-19.

PROTECT is an ongoing prospective cohort that includes >2,300 children and adolescents aged 4 months–17 years; the study monitors infections with SARS-CoV-2 in Arizona, Florida, Texas, and Utah (*4*). Children were recruited via community outreach from the public and from families participating in the HEROES-RECOVER longitudinal cohorts of essential and frontline workers (*5*,*6*). Upon enrollment, parents provided sociodemographic information, COVID-19 illness history, vaccination history, and their perceptions about COVID-19 vaccines for children. Participants are surveyed every 3 months. SARS-CoV-2 infections are identified among participant children through midturbinate nasal specimens collected weekly and tested via reverse transcription–polymerase chain reaction. Parents who completed the baseline survey and at least one follow-up survey were included in analysis. One child was randomly selected from households with two or more children aged <5 years to avoid household clustering. This study was restricted to 393 children aged <5 years who were enrolled in the PROTECT study during July 2021–May 2022. Vaccine intention was ascertained using baseline parental responses to the question, “What are the chances that [child] will get a COVID-19 vaccination?” Responses were grouped into three categories: unlikely (almost zero chance, very small chance); unsure (small chance, do not know, moderate chance); and likely (large chance, very large chance, almost certain).

A generalized estimating equation (GEE) model was used for each question to evaluate whether within-parent responses changed from a neutral or negative response (unsure or unlikely) to a positive response 3 and 6 months after the baseline enrollment survey. All available surveys from participants in the analytic group were included in the GEE models. The survey time point was added as a categorical predictor to calculate the OR for vaccine intention and vaccine perceptions. In addition, ORs describe the likelihood of all participants providing more positive responses at the 3-month and 6-month surveys compared with the baseline survey. Both unadjusted and adjusted models were calculated; the adjusted model included a positive test for SARS-CoV-2 infection in the child between surveys, sociodemographic characteristics, and study site. For vaccination intention outcomes, GEE models with multinomial distributions and cumulative logit links were used; the other models assessing perception outcomes used binomial distributions and logit links. All statistical analyses were completed using SAS (version 9.4; SAS Institute); statistical significance was defined as p<0.05 for chi-square tests and nonoverlapping 95% CIs for GEE models. PROTECT was reviewed by CDC and approved by the Institutional Review Boards at University of Arizona and Abt Associates under reliance agreements; the study was conducted consistent with applicable federal law and CDC policy.**

During July 2021–May 2022, parents provided information on 393 children aged <5 years enrolled in the PROTECT study (Table 1). The majority of children (227; 58%) resided in Arizona, and 92 (23%) had parents in the HEROES-RECOVER cohort (*5*,*6*). Median age was 2.8 years (SD = 1.3 year); 189 (48%) were male, 183 (47%) were non-Hispanic White persons, and 110 (28%) were Hispanic persons; 132 (34%) children received a positive SARS-CoV-2 test result during the study. At baseline, 253 (64.4%) parents reported that they were likely to get their child vaccinated; 76 (19.3%) were unsure, and 39 (9.9%) reported that they were unlikely to vaccinate their child (Table 1). There were statistically significant differences in vaccine intention identified by study site (p<0.001), positive SARS-CoV-2 test result during the study (p = 0.006), percent of household members vaccinated (p = 0.011), and household income (p = 0.003). 

**TABLE 1:** Baseline parental COVID-19 vaccination intent for children aged <5 years, by selected characteristics — Pediatric Research Observing Trends and Exposures in COVID-19 Timelines, four states, July 2021–May 2022

Characteristic	Participants, no. (column % or SD)	Vaccination intent, no. (row %* or SD)			p-value^†^
		Unlikely	Unsure	Likely	
**All children**	393 (100)	39 (9.9)	76 (19.3)	253 (64.4)	—
**Median age, yrs**	2.8 (1.3)	2.9 (1.3)	2.9 (1.3)	3.0 (1.1)	0.865
**Sex**					
Male	189 (48.1)	22 (11.6)	37 (19.6)	127 (67.7)	0.198
Female	186 (47.3)	16 (8.6)	38 (20.4)	126 (67.2)	
Missing	18 (4.6)	1 (5.6)	1 (5.6)	0 (—)	
**Race and ethnicity**					
White, non-Hispanic	183 (46.6)	18 (9.8)	34 (18.6)	130 (71.0)	0.400
Black, non-Hispanic	12 (3.1)	2 (16.7)	5 (41.7)	5 (41.7)	
Asian, non-Hispanic	13 (3.3)	1 (7.7)	4 (30.8)	8 (61.5)	
Hispanic	110 (28.0)	11 (10.0)	25 (22.7)	71 (64.5)	
Other	36 (9.1)	2 (5.6)	5 (13.9)	29 (80.6)	
**Site**					
Tucson, Arizona	156 (39.7)	8 (5.1)	15 (9.6)	126 (80.8)	<0.001
Phoenix, Arizona	41 (10.4)	2 (4.9)	7 (17.1)	29 (70.7)	
Other areas in Arizona	30 (7.6)	4 (13.3)	7 (23.3)	19 (63.3)	
Temple, Texas	42 (10.7)	10 (23.8)	10 (23.8)	20 (47.6)	
Salt Lake City, Utah	56 (14.3)	6 (10.7)	14 (25.0)	35 (62.5)	
Miami, Florida	68 (17.3)	9 (13.2)	23 (33.8)	24 (35.3)	
**Positive SARS-CoV-2 test result before study**	48 (12.2)	3 (6.3)	14 (29.2)	29 (60.4)	0.173
**Positive SARS-CoV-2 test result during study**	132 (33.6)	21 (15.9)	18 (13.6)	89 (67.4)	0.006
**% of household members aged >5 years vaccinated**					
0	6 (1.5)	3 (50.0)	1 (16.7)	2 (33.3)	0.011
<50	21 (5.3)	2 (9.5)	4 (19.0)	5 (23.8)	
≥50	366 (93.1)	34 (9.3)	71 (19.4)	246 (67.2)	
**Parents enrolled in adult study**	92 (23.4)	10 (10.9)	16 (17.4)	66 (71.7)	0.628
**Parent insured**					
Yes	351 (89.3)	39 (11.1)	66 (18.8)	242 (68.9)	0.026
No	23 (5.9)	0 (—)	9 (39.1)	10 (43.5)	
Missing	19 (4.8)	0 (—)	1 (5.3)	1 (5.3)	
**Household income**					
$0–$49,999	54 (13.7)	6 (11.1)	17 (31.5)	28 (51.9)	0.003
$50,000–$99,999	101 (25.7)	15 (14.9)	25 (24.8)	61 (60.4)	
$100,000–$149,999	77 (19.6)	5 (6.5)	11 (14.3)	60 (77.9)	
≥$150,000	112 (28.5)	8 (7.1)	13 (11.6)	90 (80.4)	
**Responses to vaccine questions,^§^ mean (SD)**					
Vaccination intent**^¶^**	5.7 (0.10)	1.5 (0.08)	3.7 (0.07)	6.7 (0.04)	<0.001
Chance of getting sick	4.2 (0.08)	2.6 (0.20)	3.7 (0.12)	4.6 (0.09)	<0.001
Vaccine knowledge	3.1 (0.06)	2.6 (0.14)	2.5 (0.13)	3.4 (0.07)	<0.001
Vaccine safety	3.9 (0.05)	2.5 (0.20)	3.1 (0.10)	4.2 (0.05)	<0.001
Vaccine effectiveness	3.9 (0.05)	2.6 (0.19)	3.5 (0.10)	4.2 (0.05)	<0.001
Trust in government	3.9 (0.07)	2.5 (0.21)	3.5 (0.11)	4.2 (0.08)	<0.001

* Might not sum to 100% because of rounding or missing intention category for some persons. Likely included “large chance,” “very large chance,“ and “almost certain”; unsure included “small chance,” “do not know,” and “moderate chance”; and unlikely included “almost zero chance” and “very small chance.”

^†^ Chi-square tests performed to test if the distribution of each characteristic differed by intention group. An analysis of variance was used to test if the median age of children and vaccine belief questions differed between intention groups.

**^§^** For all responses, a higher value means a more positive response. Parental perceptions toward COVID-19 vaccines were assessed with the following questions and response options: 1) “How much do you know about COVID-19 vaccines in children? Would you say…? Nothing at all, a little, some, a lot, a great deal, don’t know, decline to answer”; 2) “How safe do you think the COVID-19 vaccine is in children? Extremely safe, very safe, somewhat safe, not too safe, not at all safe, don’t know, decline to answer”; 3) “How effective do you think the COVID-19 vaccine is in preventing children from getting sick with COVID-19? Extremely effective, very effective, somewhat effective, not too effective, not at all effective, don’t know, decline to answer”; 4) “I trust what the government says about the COVID-19 vaccine. Strongly disagree, mildly disagree, neutral, mildly agree, strongly agree, don’t know, decline to answer”; 5) “What are the chances that [participant name] will get a COVID-19 vaccination? Almost zero chance, very small chance, small, moderate, large, very large chance, almost certain, don’t know, decline to answer”; and 6) “If [participant] is unable to or doesn’t get a COVID-19 vaccination, what do you think [participant]’s chance of getting sick with COVID-19 this year will be? Almost zero chance, very small chance, small, moderate, large, very large chance, almost certain, don’t know, decline to answer.”

**^¶^** This question is used to identify the vaccination intent columns.

Approximately two thirds of participants (270; 68.7%) completed a 3-month survey and 137 (34.9%) completed a 6-month survey (Table 2) (Figure). Among parents who completed a 3-month survey, 11 (4.1%) changed their vaccination intent from a neutral or negative to positive response after 3 months, although parents overall were 24% less likely to vaccinate (aOR = 0.76) than they were at baseline. Also at 3 months, 30 (11.2%) parents changed their perception of vaccine effectiveness from neutral or negative to positive, although overall, they were 39% less likely to perceive the vaccine as effective (aOR = 0.61). At 3 months after the baseline survey, perception of vaccine safety changed from neutral or negative to positive for 29 (10.9%) parents; however, overall parents were 35% less likely to perceive the vaccine as safe (aOR = 0.65). When asked about perceived trust in government, 28 (10.7%) of parents changed from a negative or neutral to a positive response after 3 months, although they were 51% less likely to report trust in the government compared with baseline (aOR = 0.49).

**TABLE 2:** Change in knowledge, attitude, and practice responses of parents of children aged <5 years from baseline to 3- and 6-month surveys — Pediatric Research Observing Trends and Exposures in COVID-19 Timelines, four states, July 2021–May 2022

Survey questions^*^/Time after baseline survey, mos.	Participant responses, no.	No. (%)		Odds ratio^†^ (95% CI)	
		Response change to neutral or negative	Response change to positive	Unadjusted	Adjusted^§^
**Intention to vaccinate**					
3	269	24**^¶^** (8.9)	11 (4.1)	0.84 (0.68–1.04)	0.76**^¶^** (0.59–0.99)
6	137	11 (8.0)	7 (5.1)	1.66**^¶^** (1.10–2.50)	1.10 (0.73–1.67)
**Chance of getting sick**					
3	270	39 (14.4)	29 (10.7)	1.16 (0.89–1.52)	1.12 (0.83–1.51)
6	135	16 (11.9)	15 (11.1)	1.40 (0.98–2.00)	1.12 (0.76–1.65)
**Vaccine knowledge**					
3	270	21 (7.8)	33 (12.2)	1.30* (1.03–1.64)	1.21 (0.93–1.58)
6	136	15 (11.0)	20 (14.7)	1.45* (1.05–2.00)	1.29 (0.88–1.88)
**Vaccine safety**					
3	266	54**^¶^** (20.3)	29 (10.9)	0.82 (0.63–1.08)	0.65**^¶^** (0.47–0.90)
6	134	7 (5.2)	17 (12.7)	1.82**^¶^** (1.29–2.57)	1.06 (0.71–1.58)
**Vaccine effectiveness**					
3	269	60**^¶^** (22.3)	30 (11.2)	0.80 (0.61–1.06)	0.61**^¶^** (0.44–0.84)
6	136	38**^¶^** (27.9)	11 (8.1)	0.76 (0.54–1.07)	0.38**^¶^** (0.25–0.57)
**Trust in government**					
3	262	65**^¶^** (24.8)	28 (10.7)	0.67**^¶^** (0.50–0.89)	0.49**^¶^** (0.34–0.71)
6	131	31**^¶^** (23.7)	11 (8.4)	1.01 (0.70–1.46)	0.51**^¶^** (0.32–0.81)

* For all vaccine perception questions except intention: odds of moving from a negative/neutral response at baseline to a positive response at follow-up. For intention: odds of being more likely to vaccinate at follow-up compared with at baseline (odds of changing from unlikely to vaccinate to being unsure of vaccinating, or unsure of vaccinating to likely to vaccinate). Odds ratio below 1 indicates less likely to go from negative/neutral to positive, and an odds ratio above 1 indicates more likely to go from negative/neutral to positive from baseline to follow-up; chance of getting sick: positive defined as “large chance,” “very large chance,” or “almost certain”; neutral defined as “small chance,” “do not know,” or “moderate chance”; and negative defined as “almost zero chance” and “very small chance”; vaccine knowledge positive defined as “a lot” or “a great deal”; neutral defined as “a little,” “some,” or “do not know”; and negative defined as “nothing at all”; vaccine safety positive defined as “very safe” or “extremely safe”; neutral defined as “somewhat safe,” “not too safe,” or “do not know”; and negative defined as “not at all safe; vaccine effectiveness positive defined as “very effective” or “extremely effective”; neutral defined as “not too effective,” “somewhat effective,” or “do not know”; and negative defined as “not at all effective”; trust in government positive defined as “strongly agree” or “agree”; neutral defined as “neutral” or “do not know”; and negative defined as “strongly disagree” or “disagree.”

^†^ Odds ratios for both time points are compared with baseline responses.

^§^Adjusted for receiving a positive SARS-CoV-2 test result before survey completion, age, sex, race and ethnicity, health insurance, and site.

**^¶^** Indicates statistically significant result.

**FIGURE:**
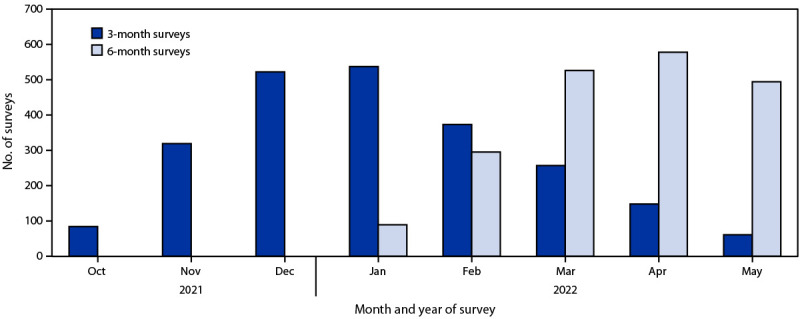
Distribution of 3-month and 6-month surveys, by study month — Pediatric Research Observing Trends and Exposures in COVID-19 Timelines cohort, four states, October 2021–May 2022

Among 137 parents who completed a 6-month survey, 11 (8.1%) changed their perception of vaccine effectiveness from neutral or negative to positive (Table 2); overall parents were 62% less likely to have a positive response (aOR = 0.38) regarding vaccine effectiveness. Eleven (8.4%) parents changed their level of trust in government from negative or neutral to positive, although overall, parents were 49% less likely to have a positive response (aOR = 0.51). In unadjusted models only, vaccination intent and perceptions of vaccine safety were less likely to be neutral or negative at 6 months (OR = 1.66 and OR = 1.82, respectively); after adjusting for receipt of a positive SARS-CoV-2 test result before 6-month survey completion, age, sex, race and ethnicity, health insurance, and site, these were no longer statistically significant.

## Discussion

Among parents of 393 children aged <5 years in this analysis, 64% indicated at baseline that they were likely to have their child vaccinated with the COVID-19 vaccine. During a 3-month observation period, however, parents indicated decreased intention to vaccinate and decreased confidence in COVID-19 vaccine safety and effectiveness as well as less trust in the government. Among the subset of participants who were in the study for 6 months, perceptions of vaccine safety, vaccine knowledge, and intent to vaccinate increased, but only in models that were not adjusted for potential confounders including SARS-CoV-2 infection during the study period. Perceptions of vaccine effectiveness and trust in government remained neutral or negative after 6 months.

The PROTECT cohort demonstrated a higher parental intent to vaccinate their young children than did other earlier surveys (*2*,*7*). Participants in COVID-19 research might be more likely than nonparticipants to comply with CDC recommendations. However, intention to vaccinate and vaccine confidence decreased over time, even though the vaccines were demonstrated to be safe and effective in older children (*8*). The decline in confidence is likely the result of multiple factors. For example, the follow-up period occurred at the time of pandemic-related events that might have affected perceptions about COVID-19 vaccines, including conflicting news reports of vaccine availability for this age group (*3*). In addition, one third of participants received positive SARS-CoV-2 test results during the observation period, which might have reduced parents’ confidence in or perceived need for the COVID-19 vaccine^††^ or reinforced assumptions of mild illness in children. Finally, news of lower estimates of vaccine effectiveness in older children potentially influenced the decline in vaccine confidence (*9*) in early 2022.

The findings in this report are subject to at least four limitations. First, follow-up surveys were distributed over 3-month periods, making discerning specific causes of changes in vaccine perception difficult. Second, because the study population is participating in a surveillance and vaccine-effectiveness study and includes frontline workers, vaccine intention might be inflated. Third, the majority of participants are from Arizona, which might not be representative of other states. Finally, not all participants in this ongoing longitudinal cohort study have been enrolled long enough to complete follow-up surveys.

This study is the first longitudinal analysis of vaccine intention and perceptions among parents of children aged <5 years. During a 3-month observation period, parents reported reduced confidence and intent to vaccinate their child when the vaccine becomes available, although their overall intent is higher than other national published rates (*2*,*7*,*10*). Enhanced efforts to identify and address parental barriers to and increase confidence in COVID-19 vaccination in children aged <5 years are needed, including educating parents about the effectiveness and safety of COVID-19 vaccination in this population.

**Summary**

**What is already known on this topic?**

In June 2022, COVID-19 vaccines were authorized for use in children aged 6 months–5 years. Intent to vaccinate and vaccination rates in children have been low.

**What is added by this report?**

During July 2021–May 2022, in a longitudinal cohort of 393 children aged <5 years in four states, parental intent to vaccinate children against COVID-19 and perception of COVID-19 vaccine safety and effectiveness declined over a 3-month period, but intent to vaccinate and perceptions of vaccine safety returned to baseline after 6 months.

**What are the implications for public health practice?**

Identifying and addressing barriers to COVID-19 vaccination in children aged <5 years and educating parents about COVID-19 vaccine effectiveness and safety in young children are critical to increasing pediatric COVID-19 vaccination coverage.

## Footnotes

* https://www.aap.org/en/pages/2019-novel-coronavirus-covid-19-infections/children-and-covid-19-state-level-data-report/ (Accessed March 9, 2022).

†
https://www.fda.gov/news-events/press-announcements/coronavirus-covid-19-update-fda-authorizes-moderna-and-pfizer-biontech-covid-19-vaccines-children

§ PROTECT is conducted in Phoenix and Tucson, Arizona; Miami, Florida; Temple, Texas; and Salt Lake City, Utah.

¶ Parental perceptions toward COVID-19 vaccines were assessed with the following questions and response options: 1) “How much do you know about COVID-19 vaccines in children? Would you say…? Nothing at all, a little, some, a lot, a great deal, don’t know, decline to answer”; 2) “How safe do you think the COVID-19 vaccine is in children? Extremely safe, very safe, somewhat safe, not too safe, not at all safe, don’t know, decline to answer”; 3) “How effective do you think the COVID-19 vaccine is in preventing children from getting sick with COVID-19? Extremely effective, very effective, somewhat effective, not too effective, not at all effective, don’t know, decline to answer”; 4) “I trust what the government says about the COVID-19 vaccine. Strongly disagree, mildly disagree, neutral, mildly agree, strongly agree, don’t know, decline to answer”; 5) “What are the chances that [participant name] will get a COVID-19 vaccination? almost zero chance, very small chance, small, moderate, large, very large chance, almost certain, don’t know, decline to answer”; and 6) “If [participant name] is unable to or doesn’t get a COVID-19 vaccination, what do you think [participant name]’s chance of getting sick with COVID-19 this year will be? Almost zero chance, very small chance, small, moderate, large, very large chance, almost certain, don’t know, decline to answer.”

** 45 C.F.R. part 46; 21 C.F.R. part 56; 42 U.S.C. Sect. 241(d), 5 U.S.C. Sect. 552a, 44 U.S.C. Sect. 3501 et seq.

††
https://www.kff.org/coronavirus-covid-19/poll-finding/kff-covid-19-vaccine-monitor-april-2022
